# Stability and change of lifestyle profiles in cardiovascular patients after their first acute coronary event

**DOI:** 10.1371/journal.pone.0183905

**Published:** 2017-08-29

**Authors:** Patrizia Steca, Dario Monzani, Andrea Greco, Cristina Franzelli, Maria Elena Magrin, Massimo Miglioretti, Marcello Sarini, Marta Scrignaro, Luca Vecchio, Francesco Fattirolli, Marco D’Addario

**Affiliations:** 1 Department of Psychology, University of Milan –Bicocca, Milan, Italy; 2 Cardiac Rehabilitation Centre, Istituti Clinici di Perfezionamento Hospital, Milan, Italy; 3 Department of Medical and Surgical Critical Care, Cardiac Rehabilitation Unit, University of Florence and Azienda Ospedaliero-Universitaria Careggi, Florence, Italy; University of Bologna, ITALY

## Abstract

**Background:**

Acute coronary syndrome (ACS) is a major cause of morbidity and mortality. Lifestyle and health behavior changes play an important role in the primary and secondary prevention of ACS recurrence. Changes in unhealthy lifestyles after an acute coronary event have been analyzed by considering separate behaviors individually, even though research on the healthy population has demonstrated that unhealthy behaviors tend to co-occur.

**Purpose:**

The aim of this study was to identify lifestyle profiles of ACS patients and to explore their pathways of change for one year after their first coronary event by adopting a typological approach.

**Methods:**

Two hundred and twenty-three patients (84% male; mean age = 57.14) completed self-report measures of health-related behaviors at the beginning of cardiac rehabilitation, and six months and twelve months after. At each wave depression, anxiety and heart rate were also evaluated. Cluster analysis was performed to identify lifestyle profiles and to analyze their change over time. Differences in psychological factors and heart rate among clusters were assessed.

**Results:**

Patients' diet, physical activity, and smoking behavior greatly improved six months after their first coronary event. No further improvements were detected after one year. At each wave specific lifestyle profiles were identified, ranging from more maladaptive to healthier clusters. Patients with multiple unhealthy behaviors experience greater difficulties in maintaining a healthier lifestyle over time. Moreover, the results demonstrated the association between lifestyle profiles at twelve months after the acute coronary event and depression measured six months earlier. Finally, the most maladaptive lifestyle profile had many members with elevated heart rate at twelve months after the cardiac rehabilitation.

**Conclusions:**

Current findings may have a strong practical impact in the development and implementation of personalized secondary prevention programs targeting lifestyles of ACS patients.

## Introduction

Acute Coronary Syndrome (ACS) is still the most common cause of morbidity and mortality in Western countries [1–2]. Although there has been an appreciable improvement in the treatment of ACS, the risk of subsequent cardiovascular events and cardiovascular mortality is still high. Specifically, a recent French study attested that total 1-year mortality is 29.3% [3]. Moreover, recurrences at six and twelve months are about 23% and 36%, respectively [4]. The treatment of ACS and subsequent cardiovascular events represents a major, global economic burden for healthcare systems. It is thus essential to promote effective secondary prevention in order to improve the prognosis of patients with ACS.

The study of the varying degrees of co-occurrence among lifestyle risk factors may play a pivotal role in the secondary prevention of this clinical condition. First, similarly to other diseases [5–6], lifestyle risk factors may have synergistic and multiplicative health effects rather than an additive effect in ACS patients. Second, evidence of varying degrees of co-occurrence of lifestyle risk factors may better direct more effective behavioral change interventions for the secondary prevention of ACS. Among lifestyle risk factors, cardiac rehabilitation for secondary prevention of ACS mainly focuses on the promotion of healthy behavioral change in terms of quitting smoking, adopting healthy eating habits and being physically active [7]. Nevertheless, the recent European Action on Secondary and Primary Prevention by Intervention to Reduce Events IV [8] pointed out that a small proportion of patients are unable to effectively achieve the healthy lifestyle targets after hospitalization for ACS.

Empirical evidence has suggested that the clustering of lifestyle risk factors for cardiovascular diseases exists in the healthy population, where unhealthy lifestyles are not randomly distributed, but tend to co-occur in combination with other behavioral risk factors [9–11]. For example, some studies have suggested associations between physical activity and healthy eating habits [12], smoking and eating habits [13], and smoking and physical exercise [14].

No study has investigated the clustering of unhealthy lifestyles in ACS patients at their first event, namely at a point in their lives where they face a shocking event and receive multiple demands to change their lifestyles. Moreover, no study has investigated how lifestyle clusters improve or worsen over time. Evaluating the co-occurrence and change of lifestyle risk factors in ACS patients may direct the implementation of personalized programs targeting health related behaviors and this may have a great impact on the effectiveness of healthcare practices. Knowledge on how behaviors change over time may further help to personalize interventions and make them more effective.

The aim of this study was to identify clusters of lifestyle risk factors in ACS patients before their first coronary event and to analyze their changes six and twelve months later by adopting a typological approach. Specifically, during cardiac rehabilitation (T0), retrospective measures of behaviors regarding diet, physical activity, and cigarette smoking were obtained. The use of retrospective measures makes it possible to analyze the change in lifestyle clustering from the period before the acute coronary event to six (T1) and twelve months after it (T2). At these two later time points, concurrent measures of diet, physical activity and smoking were considered. At each wave measures of depression, anxiety, and Heart Rate (HR) were also collected. At T1 and T2 differences in preceding measures of depression and anxiety among lifestyle clusters were assessed to explore the possible association of these psychological variables with subsequent behavioral change. Depression and anxiety have been selected for their significant associations to ACS onset and recurrence [15–16], and for their effect on patients’ likelihood to adhere to lifestyle modification programs and then to effectively quit smoking, adopt a healthier diet and be more physically active [17–20]. At each wave the associations between cluster membership and HR were also explored. HR is an important prognostic factor in cardiovascular disease and studies on the specific population of ACS patients have shown a significant link between elevated HR (i.e., ≥ 70 bpm) and the risk of subsequent cardiovascular deaths and morbidity [21].

In reaching its goals the current study aimed to elucidate the following specific research questions:

1. Which lifestyle profiles, defined as clusters, characterize ACS patients before their first acute event, and then six and twelve months after this event? How do the relative frequencies of healthier and maladaptive lifestyle clusters change from before the event to six and twelve months after it? Based on empirical evidence from the general population, we hypothesized finding multiple lifestyle clusters varying in degrees of healthiness, from maladaptive, unhealthier clusters to healthier profiles. No specific hypotheses were advanced as regards specific associations among health behaviors due to the heterogeneous results reported in the literature cited above. Moreover, recent empirical findings of the European Action on Secondary and Primary Prevention by Intervention to Reduce Events IV [8] showed that, by considering each individual behavior at a time, ACS patients generally report an improvement in the healthiness of their lifestyles. Following this study, we hypothesized that, by adopting a typological approach, frequencies of maladaptive clusters tend to decrease and healthier clusters tend to increase over time, reflecting an overall improvement in the healthiness of lifestyles after the coronary event.
2. What are the individual pathways of change in lifestyle cluster membership from T0 to T1 and from T1 to T2? In line with recent findings [8] and our previous hypothesis, we expected a moderate improvement of healthy behavior. Specifically, we hypothesized that patients with unhealthier lifestyle profiles before the acute event would be more likely to be in a healthier cluster six and twelve months later. At the same time, we expected that patients with a more positive lifestyle profile would tend to remain in the same cluster across the time period considered.
3. What are the differences among lifestyle profiles in preceding measures of depression and anxiety? Following evidence suggesting a deleterious effect of psychological distress on overall behavioral change [17–20], we expected that unhealthier lifestyle profiles would be characterized by higher levels of antecedent measures of depression and anxiety.
4. At each wave, which are the associations between cluster membership and HR cutoff? Following evidence of the detrimental influence of unhealthy lifestyles on the autonomic nervous systems, as shown by elevated HR in people with low physical activity, heavy smoking, and inadequate diet [22–23], we expected an association between maladaptive cluster membership and elevated HR.

## Method

### Participants and procedure

Two hundred and seventy-two consecutive ACS patients at their first coronary event were recruited for the current study in three Italian hospitals: Azienda Ospedaliero-Universitaria Careggi of Florence, Istituti Clinici di Perfezionamento Hospital of Milan, and Azienda Ospedaliera Bolognini of Seriate. They were recruited from February 2011 to October 2013. Patients who were eligible to participate in the study were between 30 and 80 years of age and had sufficient Italian language skills. Patients with cognitive deficits or other major pathologies (such as cancer) were excluded. They were recruited during their CR at the hospital, which took place between two and eight weeks after their acute coronary event. Attrition rates were low; 12.5% of patients were absent at T1 and 14.7% were absent at T2. Principal causes of attrition included: emigration, refusal and, in a small minority of cases, the patient was untraceable.

Almost all patients were prescribed pharmacological treatment for ACS, consisting of antiplatelet drugs (99% of patients), beta-blockers (89%), statins (97%), sartans or ace-inhibitors (99%). Results of paired McNemar tests attested that pharmacological prescription did not change over time (i.e., T1: antiplatelet drugs: 100%; beta-blockers: 90%; statins: 97%; sartans or ace-inhibitors: 100%; T2: antiplatelet drugs: 98%; beta-blockers: 87%; statins: 95; sartans or ace-inhibitors: 99%).

Because the typological data analyses we adopted use only manifest data and do not accommodate for missing values, analyses were performed on 187 male (84%) and 36 female (16%) patients with complete information across the three waves. The proportion of males in this sample was a direct consequence of the incidence of ACS, which is more common among men than women. The mean age was 57.14 years (*SD* = 8.00, range: 34–77 years). The research was approved by the Ethics Committee at the authors’ University and at each hospital involved. After an informal agreement with the patient, the physician introduced the researcher who then asked the participant to read information about the study and sign the written informed consent form before completing a set of self-report questionnaires.

### Measures

At each wave, self-report instruments were administered by a trained researcher to assess healthy behaviors regarding diet, physical activity, and cigarette smoking and to assess anxiety and depression. At T0, patients were asked to think about their nutrition, physical activity and smoking behavior before the coronary event, whereas at the two subsequent follow-ups they were asked to report their behavior at that time. HR measurements were collected by physicians through a standard electrocardiogram test in a clinical setting with patient resting five minutes in supine position.

#### Diet

To measure dietary behavior, the Italian version of the Mediterranean Diet Scale [24–25] was used. The instrument is a 9-item questionnaire that measures the weekly consumption of nine foods using a 6-point Likert scale (from 1 = Never to 6 = More than three times per day). The consumption of both beneficial (i.e., vegetables, fruits, whole grains, fish, legumes, olive oil) and detrimental foods (i.e., more than two glasses of wine per day for men and more than one glass of wine for women, butter and margarine or vegetable oil other than olive oil, red or processed meat) was assessed. The sum of the recoded responses yielded the Mediterranean Diet Score, on which higher scores indicated a healthier diet. A score of four and above shows good adherence to the Mediterranean diet (MD) and has been related to good health outcomes [26].

#### Physical activity

Physical activity was measured by the Rapid Assessment of Physical Activity Questionnaire-1 [27]. It is a 7-item questionnaire that measures the type and amount of reported physical activity using a yes/no dichotomous scale and covers the range of levels of physical activity from sedentary to regular vigorous physical activity. The total score ranged from one (i.e., sedentary) to seven (i.e., regular and vigorous active), with higher scores indicating a healthier amount of physical activity. Moreover, patients could be considered active when: (1) doing 30 minutes or more a day of moderate physical activity, five or more days a week, or (2) doing 20 minutes or more a day of vigorous physical activity, three or more days a week.

#### Cigarette smoking behavior

Participants’ smoking behavior was measured with the question “How many cigarettes do you smoke per day?”; the scale ratings were 0 = “No cigarettes”, 1 = “10 cigarettes or fewer per day”, 2 = “11–20 cigarettes per day”, 3 = “21–30 cigarettes per day”, and 4 = “31 or more cigarettes per day”. In the present research, this total score was reversed to reflect higher scores representing healthier smoking behavior.

#### Anxiety and depression

We used the Italian version of the Hospital Anxiety and Depression Scale [28–29], a 14-item self-report measure developed to screen for generalized symptoms of depression and anxiety in medical patients. Participants reported their feelings and moods on a four-point Likert scale; a sample item from this instrument is “I’ve lost interest in my appearance” (possible answers are: 3 = definitely; 2 = I don’t take as much care as I should; 1 = I may not take quite as much care; 0 = I take just as much care as ever). Two sum scores are calculated for anxiety and depressive symptoms; their total scores range from 0 to 21, in which higher scores indicate greater presence of mood disorders.

### Data analysis

Data analyses were performed with SPSS and specific modules of Sleipner [30], a statistical package for typological analyses.

#### Cluster analysis and identification of lifestyle profiles

Cluster analyses were performed on the continuous scores of the three lifestyle variables. Specifically, these analyses were performed at each wave separately. To perform each cluster analysis, we followed the suggestion of Bergman [31]. In the first step, all three lifestyle variables were z-standardized at each wave separately. In this second step, before each cluster analysis, we performed a residue analysis according to standard options [32] (i.e., Average Squared Euclidean Distance (ASED) less than .5) we identified three multivariate outliers at T1 and seven at T2 and then excluded them from the subsequent analysis; no outlier was identified at T0. Subsequently, a two-step clustering procedure was applied at each wave separately to typify patients depending on their behaviors. A combination of hierarchical and non-hierarchical clustering methods was used. Ward’s hierarchical method, followed by the non-hierarchical k-means method, was used. In the hierarchical method, various solutions were chosen based on the size of the change in the error sum of square (ESS) value between adjacent cluster solutions. Therefore, each solution was subsequently used as the initial cluster center for a non-hierarchical k-means clustering procedure. After this non-hierarchical clustering method, four indices were used to evaluate the optimal number of clusters to extract at each wave: the C-index, the G(+) index, the Gamma index, and the Point biserial correlation. The minimum value of the former two indices and maximum of the latter two suggested the optimal number of clusters to retain, hence the best cluster solution. Another criterion for cluster solution retention was a reasonable cluster size (i.e., every cluster contained at least 5% of all the cases) [33]). An analysis was performed on each wave separately: the decision made for the analysis of one measurement point did not influence decisions made for the other two waves. At this stage, Pearson’s Chi-squared tests were performed to evaluate the association of cluster membership at each wave with the participants’ gender. Similarly, at each wave an ANOVA was performed to assess age difference across lifestyle clusters.

#### Individual pathways

Individual pathways of change were evaluated by studying individual movements between clusters at different waves. Specifically, by following the guidelines by Bergman [32], we identified individual pathways by performing exact analysis of single cells in a contingency table for two categorical variables (e.g., cluster membership at T0 vs. cluster membership at T1; cluster membership at T1 vs. cluster membership at T2). This procedure focuses on cell-wise analysis of types based on exact tests. Specifically, a significant individual pathway is said to occur in a cell if its observed frequency is much larger than expected and the associated hypergeometric probability is low. In other words, this analysis evaluates the T0-to-T1 and the T1-to-T2 sequences of lifestyle profile to verify whether these sequences occur more often than expected by chance.

#### Differences in psychological factors and HR

At T1 and T2, MANOVAs were performed to assess the differences among the lifestyle profiles identified in the preceding measures of depression and anxiety. Specifically, while at T1 we performed a MANOVA analyzing the difference in psychological distress at T0, at T2 we executed two MANOVAs assessing differences in psychological distress at T0 and T1. Moreover, at each wave, Pearson’s Chi-squared test was performed to evaluate the association of cluster membership with HR cut-off (i.e., Low risk: HR< 70 bpm; High risk: HR≥ 70 bpm). Similarly, we evaluated the difference in psychological factors and distribution of HR cut-off at T2 among identified individual pathways over time.

## Results

### Identification of lifestyle profiles

After an evaluation of the scree-type plots showing the change in ESS by cluster solutions and based on the size of the change in the ESS values, the solutions from the five- to six-clusters at T0 were retained for further analysis. At T1, the solutions from four- to six-clusters were retained. Finally, at T2 the scree plots suggested retaining the solutions from five- to seven-cluster solutions. Table 1 presents the fit indices of the retained cluster solutions at each wave. At T0, even though the C-index is lower and the Point biserial correlation is higher in the five-cluster solution, the G(+) index and the Gamma index are more appropriate in the solution with six-clusters. However, the five-cluster solution was preferred due to the parsimony principle and theoretical meaningfulness. At T1, only the Point biserial correlation favored the four-cluster solution. Thus, the solution with six-clusters was preferred because its C-index, Gamma index and G(+) are the most appropriate. Finally, at T2 the solution from six- to seven-clusters could not be considered because they were composed of very small clusters with fewer than 5% of all the cases. Thus, the solution with five-clusters was chosen.

**Table 1:** Fit indices of cluster solutions identified through k-means cluster analysis at each wave.

	T0		T1			T2		
	#5	#6	#4	#5	#6	#5	#6	#7
C-index	.15	.18	.11	.08	.06	.05	.10	.11
G(+) index	.04	.03	.05	.03	.02	.03	.03	.02
Gamma index	.75	.80	.76	.81	.86	.83	.85	.87
Point biserial correlation	.43	.41	.47	.41	.40	.42	.43	.41
ESS	64.52	70.92	61.23	7.12	75.66	71.49	76.53	79.62

*Note*. ESS = explained error sum of squares of the given classification.

**Fig 1:**
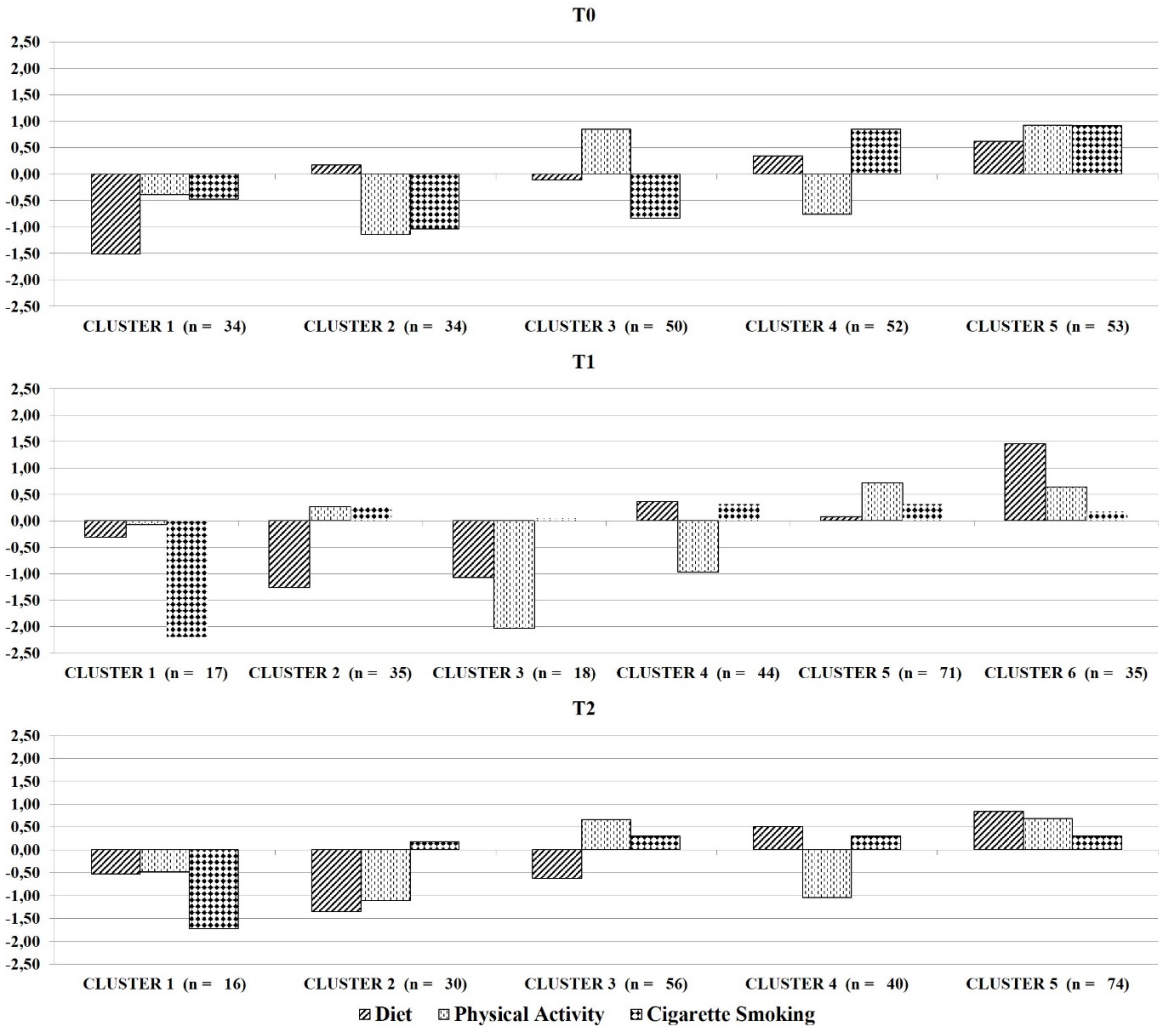
Lifestyle profiles characterized by their z-scores for diet, physical activity, and cigarette smoking behaviors for each of the three waves.

Fig 1 displays the mean standardized scores (i.e., *z*-scores) of the diet, physical activity, and cigarette smoking behavior indices for the five-, six- and five-cluster solutions identified at T0, T1, and T2, respectively. In this representation *z*-scores between– 0.5 and + 0.5 denote an average value (i.e., the “average ACS patient” lifestyle). Z-scores under– 0.5 represent values below the sample mean (i.e., less healthy than average lifestyle), lower adherence to the MD, lower physical activity, and heavier smoking than the “average ACS patient”. *Z*-scores over + 0.5 denote values above the sample mean (i.e., healthier than average lifestyle) thus, higher adherence to the MD, higher physical activity, and lighter smoking than the “average ACS patient” does.

Table 2 reports a socio-demographic description and adequateness of lifestyle for the identified clusters at each wave. At T0 only 20.6% of patients had an adequate diet, 36.3% were physically active and 39.5% were non-smokers. Five lifestyle profiles were identified. *Cluster 1* (N = 34; 15% of the classified lifestyle profiles at T0; mean age = 52.97 years; 82.4% male) was characterized by lower adherence to the MD than the average ACS patient before the acute coronary event. No patient belonging to this cluster adhered to the MD; only 8.8% were physically active and 8.8% were non-smokers. *Cluster 2* (N = 34; 15% of the classified lifestyle profiles at T0; mean age = 57.91 years; 88.2% male) was characterized by lower physical activity and heavier smoking than average. None of the patients were physically active and non-smoking, whereas only 11.8% adhered to the MD. This was the most maladaptive lifestyle profile at the first wave, followed by Cluster 1. *Cluster 3* (N = 50; 23% of the classified lifestyle profiles at T0; mean age = 54.06 years; 75.0% male) was a profile of heavy smoking (0% of the patients were non-smokers) but higher physical activity than average (72.0% were physically active). As regards diet, only 10.0% of patients adhered to the MD. *Cluster 4* (N = 52; 23% of the classified lifestyle profiles at T0; mean age = 59.42 years; 75.0% male) could be described as having lower physical activity (no patient was physically active) but lighter smoking (76.9% of patients were non-smokers). Diet behavior was similar to the average ACS patient (26.9% in this cluster adhered to the MD). *Cluster 5* (N = 53; 24% of the classified lifestyle profiles at T0; mean age = 60.2 years; 84.9% male) was characterized by higher adherence to the MD, higher physical activity and lighter smoking profile. This was the healthiest lifestyle profile at T0, with 43.9% of patients in the cluster adherent to the MD, 79.2% physically active, and 84.9% non-smoking. The result of a Chi-squared test showed no gender difference across these lifestyle profiles [*Χ*^*2*^(4; N = 223) = 4.99; *p* = .288]. However, the result of a one-way ANOVA revealed that clusters differ in age [*F*(4, 217) = 7.86, *p* = .000]; specifically, a Bonferroni post-hoc test highlighted that Clusters 1 and 3 were significantly younger than Clusters 4 and 5.

**Table 2:** Sociodemographic description and adequateness of lifestyle for the identified clusters at each wave.

Wave	Clusters	N	% at each wave	Mean age (*SD*)	% male	% adherent to MD	% physically active	% non-smoking
**T0**	#1	34	15	52.97 (9.25)	82.4	0	8.8	8.8
	#2	34	15	57.91 (7.09)	88.2	11.8	0	0
	#3	50	23	54.06 (6.76)	90.0	10.0	72.0	0
	#4	52	23	59.42 (7.97)	75.0	26.9	0	76.9
	#5	53	24	60.02 (6.84)	84.9	43.4	79.2	84.9
	Total	223		57.14 (8.00)	83.9	20.6	36.3	39.5
**T1**	#1	17	8	56.06 (8.59)	94.1	23.5	41.2	0
	#2	35	16	57.91 (7.99)	88.6	0	65.7	97.1
	#3	18	8	57.72 (7.74)	72.2	5.6	0	77.8
	#4	44	20	58.72 (6.79)	75.0	68.2	0	100
	#5	71	32	57.13 (8.44)	83.1	50.7	100	98.6
	#6	35	16	58.21 (8.77)	91.4	100	97.1	91.4
	Total	220		57.71 (8.01)	83.6	48.2	61.4	88.2
**T2**	#1	16	7	53.75 (8.79)	81.3	25.0	37.5	0
	#2	30	14	57.37 (8.09)	75.9	0	0	89.7
	#3	56	26	59.05 (8.42)	92.9	0	92.9	100
	#4	40	19	61.75 (5.83)	80.0	70.0	0	100
	#5	74	34	56.88 (7.99)	82.4	97.3	95.9	97.3
	Total	216		58.19 (8.05)	83.7	48.4	60.0	90.2

*Note*: MD = Mediterranean diet.

At T1, behaviors were greatly improved: 48.2% of patients had an adequate diet, 61.4% were physically active and 88.2% were non-smokers. Six lifestyle profiles were identified. *Cluster 1* (N = 17; 8% of the classified lifestyle profiles at T1; mean age = 56.06 years; 94.1% male) was one of the worst clusters at T1 and was characterized by heavier smoking than average. All patients were smokers, whereas only 23.5% adhered to the MD and 41.2% were physically active. *Cluster 2* (N = 35; 16% of the classified lifestyle profiles at T1; mean age = 57.91 years; 88.6% male) was characterized by lower adherence to the MD than average (0% adhered). Patients in this cluster were close to the average as regards physical activity (65.7% were active) and smoking behavior (97.1% were non-smokers). *Cluster 3* (N = 18; 8% of the classified lifestyle profiles at T1; mean age = 57.72 years; 72.2% male) was the most maladaptive lifestyle profile at the second wave, characterized by lower adherence to the MD and lower physical activity than average. None of the patients belonging to this cluster was physically active and only 5.6% of them adhered to the MD. As regards smoking 77.8% of patients were non-smokers. *Cluster 4* (N = 44; 20% of the classified lifestyle profiles at T1; mean age = 58.72 years; 75.0% male) was described as having lower physical activity (0% physically active) and not smoking (100.0% non-smokers). Their diet behavior was slightly better than the average ACS patient (68.2% adhered to the MD). *Cluster 5* (N = 71; 32% of the classified lifestyle profiles at T1; mean age = 57.13 years; 83.1% male) had a higher physically active profile (100.0% active). The diet behavior and smoking of patients in this cluster were close to average (50.7% adhered to the MD; 98.6% non-smoker). The last cluster, named *Cluster 6* (N = 35; 16% of the classified lifestyle profiles at T1; mean age = 58.21years; 91.4% male), was the healthiest lifestyle profile at T1, with all patients in the cluster adherent to the MD, 97.1% physically active, and 91.4% non-smoking. Compared to the average ACS patient, this cluster adhered more to the MD and was more physically active six months after the acute coronary event. The result of a Chi-square test showed no gender difference across these lifestyle profiles [*Χ*^*2*^(5; N = 220) = 7.67; *p* = .176]. Moreover, the result of a one-way ANOVA revealed no cluster differences in age [*F*(5, 213) = .39, *p* = .858].

At T2 lifestyle indicators were similar to T1, reflecting behavioral stability in the whole group of patients: 48.4% of patients had an adequate diet, 60% were physically active and 90.2% were non-smokers. Five lifestyle profiles were identified. *Cluster 1* (N = 16; 7% of the classified lifestyle profiles at T2; mean age = 53.75 years; 81.3% male) was characterized by lower adherence to the MD (25.0% adhered) and heavier smoking (0% non-smokers) than the average ACS patient. Also the percentage of physically active patients was low (37.5% active). *Cluster 2* (N = 30; 14% of the classified lifestyle profiles at T2; mean age = 57.37 years; 75.9% male) was characterized by lower adherence to the MD (0% adhered) and lower physical activity (0% was active) than the average. These patients’ smoking behaviors were similar to the average (89.7% were non-smokers). These two clusters were the most maladaptive lifestyle profiles at the third wave, twelve months after the coronary event. *Cluster 3* (N = 56; 26% of the classified lifestyle profiles at T2; mean age = 59.05 years; 92.9% male) was a profile of lower adherence to the MD (0% adherent) but higher physical activity (92.9% active) than average. All patients were non-smokers. *Cluster 4* (N = 40; 19% of the classified lifestyle profiles at T2; mean age = 61.75 years; 80.0% male) could be described adhering more to the MD but lower physical activity than the average. In this cluster 70.0% of patients adhered to the MD, whereas none was physically active. Similar to the previous cluster all patients were non-smokers. *Cluster 5* (N = 74; 34% of the classified lifestyle profiles at T2; mean age = 56.88 years; 82.4% male), which adhered more to the MD and was more physically active, was the healthiest lifestyle profile at T2; 97.3% of patients belonging to this cluster adhered to the MD, 95.9% were physically active, and 97.3% were non-smokers. The result of a Chi-square test showed no gender difference across these profiles [*Χ*^*2*^(4; N = 216) = 5.31; *p* = .257]. However, the result of a one-way ANOVA revealed that clusters differed in age [*F*(4, 209) = 4.12, *p* = .003]; specifically, a Bonferroni post-hoc test highlighted that Cluster 4 was significantly older than Clusters 1 and 5.

### Individual pathways

**Fig 2:**
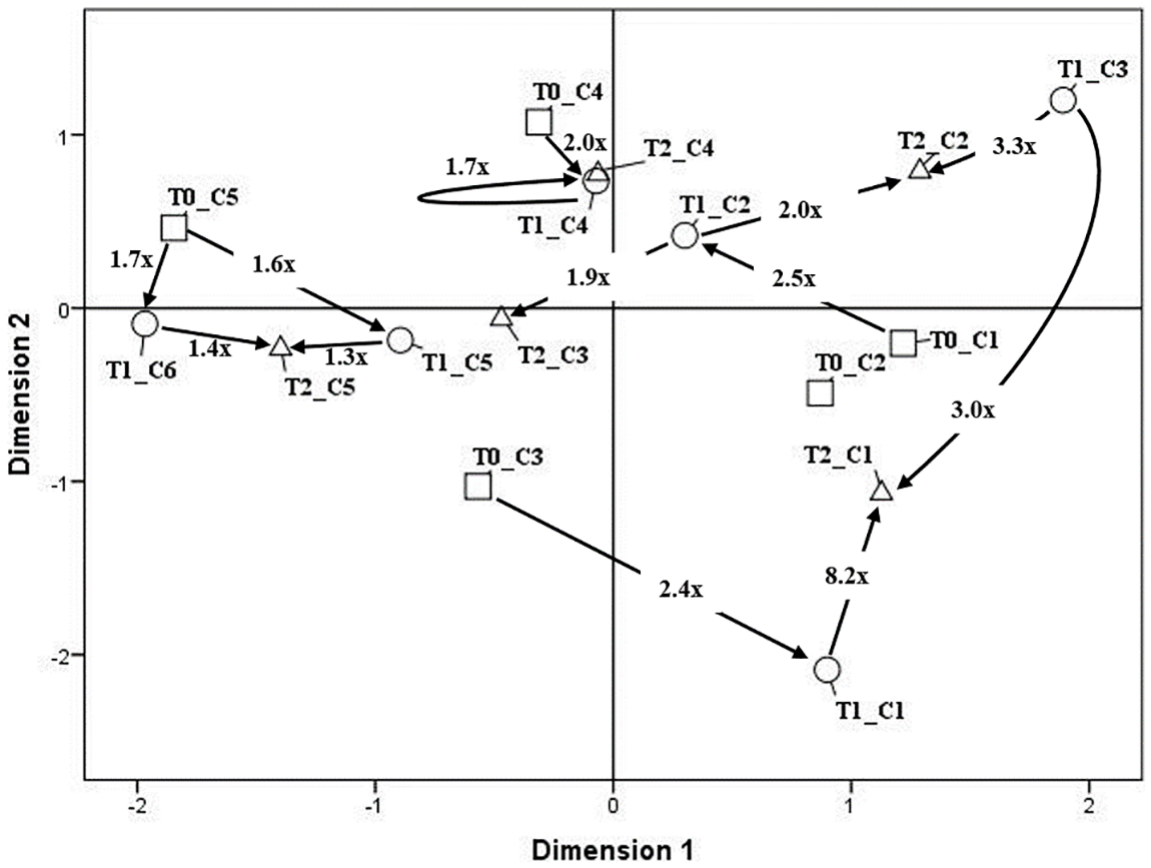
Two-dimensional map for the individual pathways across the three waves. Arrows indicated significant individual pathways (i.e., *p*< .05). Numbers at arrows indicated the ratio between observed and expected frequency of each significant pathway. T0_ denotes clusters at T0; T1_ denotes clusters at T1; T2_ denotes clusters at T2.

Fig 2 is a two-dimensional map graphically representing dissimilarities between lifestyle profiles and the individual pathways from T0-to-T1 and T1-to-T2. This two-dimensional map was obtained by carrying out multidimensional scaling in which the Euclidean distance between two lifestyle profiles was represented by the spatial distance between them and by extracting a two-dimensional solution with good fit (Young’s S-Stress = .15). Thus, in this figure the arrows represent significant individual pathways, whereas the numbers on the arrows represent the ratio between the observed and the expected frequency. Specifically, significant individual pathways were identified by considering the results of the Fisher’s hypergeometric distribution test computed with Sleipner. As shown, we identified five T0-to-T1 developmental pathways that are followed more often than expected by chance. Three out of five of these pathways could be defined as large changes in lifestyle profile since the two-dimensional map suggested that in both cases the pair of clusters was rather dissimilar and distant to each other (i.e., ASED> .25) [31]. Specifically, the sequence from the first lifestyle profile at T0 to the second at T1 occurred 2.5 times more than expected under an independence model. These two clusters are separated by .35 ASED. This pathway is characterized by a great improvement in physical activity and smoking behavior associated to the complete stability of the unhealthy diet (no patient in either cluster adhered to the MD). A similar distance (i.e., .45) separated the fifth cluster at T0 from the sixth lifestyle profile at T1. This sequence is characterized by a great improvement in diet: 43.4% of patients adhered to the MD in the fifth cluster at T0, whereas 100% adhered at T1. A larger distance (i.e., ASED = .91) separated the third cluster at T0 from the first lifestyle profile at T1. This pathway is characterized by severe worsening in physical activity (from 72% to 41.2% of active patients) and stably unhealthy diet and smoking (all patients were smokers in both clusters). The remaining two T0-to-T1 pathways were between pairs of clusters that were very similar to each other. An ASED of .11 separated the fourth cluster at T0 from the fourth cluster at T1; both clusters are characterized by insufficient physical activity. An ASED of .23 separated the fifth lifestyle profile at T0 from the fifth profile at T1. Both clusters were the healthiest in their respective waves. Because of this closeness, these individual pathways could be defined as patterns of individual stability.

By considering individual change in cluster membership across the second and third wave, results underlined eight significant individual pathways. Three out of eight of these pathways could be defined as great changes in lifestyle profile. An individual pathway existed between the second lifestyle profile at T1 and the second at T2; these two clusters are separated by .64 ASED. This sequence is characterized by stark worsening in physical activity (from 65.7 to 0% of active patients) and a stably unhealthy diet (no patient adhered to the MD in either cluster). A notable distance (i.e., .31) separated the third cluster at T1 from the second lifestyle profile at T2. This pathway is characterized by slight worsening in diet and a slight improvement in smoking, whereas physical activity remains stably insufficient. The broadest distance (i.e., ASED = 1.95) separated Cluster 3 at T1 from the first lifestyle profile at T2. This sequence is characterized by a slight improvement in adherence to the MD and physical activity, but by stark worsening in smoking behavior (from 77.8% to 0% of non-smokers)

The remaining five T1-to-T2 pathways were between pairs of clusters that were very similar to each other (i.e., Cluster 6-to-Cluster5; Cluster 4-to-Cluster 4; Cluster 1-to-Cluster 1; Cluster 2-to-Cluster 3; Cluster 5-to-Cluster 5). Because of this closeness, these individual pathways could be defined as patterns of individual stability. The sequence ‘Cluster 6-to-Cluster 5’ reflects stably positive conduct as both clusters were the healthiest in their respective waves. The sequence 'Cluster 4-to-Cluster 4' is characterized by stably insufficient physical activity (no patient was active in either cluster), whereas the pathway 'Cluster 2-to-Cluster 3' reflects a stably unhealthy diet (no patient adhered to the MD in either cluster). Finally, the sequence 'Cluster 5-to-Cluster 5' is characterized by an improvement in healthy diet (from 50.7% to 97.3% of adherence to the MD) and stable physical activity and non-smoking behavior.

### Differences in depression, anxiety, and HR

A series of MANOVAs were performed to assess differences at T1 and T2 in preceding measures of psychological distress. Both gender and age differences were introduced as covariates to control for their effect. Results demonstrated a significant effect of cluster membership at T2 on the combined variable of psychological distress at T1 [*F* (8, 412) = 2.14; *p* = .031; Wilk’s Lambda = .92]. Analysis of each individual dependent variable showed that clusters differed in depression [*F* (4, 207) = 3.87, *p* = .005] but not in anxiety [*F* (4, 207) = 1.95, *p* = .104]. As revealed by Bonferroni’s post-hoc test, patients classified in the first cluster reported higher preceding measures of depression at T1 (*M* = 6.39; *sd* = 0.75) compared to Cluster 3 (*M* = 3.62; *sd* = 0.45) and Cluster 5 (*M* = 4.10; *sd* = 0.38). The results of the two MANOVAs assessing differences in psychological distress at T0 among cluster membership at T1 and T2 did not reveal a significant effect [T1: *F* (10, 420) = 1.57; *p* = .112; Wilk’s Lambda = .93; T2: *F* (8, 412) = 1.90; *p* = .058; Wilk’s Lambda = .93].

The analysis of the association of cluster membership at each wave with concurrent measures of HR revealed that at T2 there was a relationship between HR cut-off and cluster membership [*Χ*^*2*^(4; N = 204) = 5.31; exact *p* = .009]. Specifically, results underlined a predominance of high-risk HR in the second lifestyle profile characterized by lower adherence to the MD and lower physical activity.

## Discussion

The present study is the first attempt to identify clustering of lifestyle risk factors in ACS and to analyze their changes six and twelve months after their first coronary event by adopting a typological approach. Consistent with our hypotheses and with the results from the general healthy population [9–11, 14], our findings have demonstrated the co-occurrence and interrelatedness of adhering to the Mediterranean diet, engaging in physical activity and smoking in ACS patients. At each wave, we identified different behavioral profiles, varying in healthiness from riskiest and most maladaptive to healthier profiles. Specifically, only one identified cluster at T0 and T2 and two clusters at T1 can be labeled as more adaptive and lower risk clusters because each of them is characterized by the absence of or lower degrees of dysfunctional behavioral habits. The percentage of good behaviors in each cluster is quite different moving from baseline to T2, one year after the event. Whereas in the healthiest cluster at T0 a majority of patients were physically active, non-smokers, and around 40% adhered to the Mediterranean diet, the profiles were more positive at T1 and T2, where almost all patients behave properly, especially as regards physical activity and smoking. These findings suggest a general improvement of health-related habits six months after the acute event. No further improvement was detected twelve months later, as clearly confirmed by the percentages of proper behaviors in the entire group, which greatly improved from baseline to T1 and then remained stable at T2. Considering that only 60% of patients were physically active and less than 50% ate properly at the final assessment, our results confirm the difficulty that patients experience in changing their lifestyle, especially as regards diet. The great difficulty in changing unhealthy nutrition after ACS is also attested by a recent qualitative analysis of coronary heart disease patient views of dietary adherence [34]. This study pointed out that two of the largest challenges were reducing carbohydrate intake and portion control. Moreover, many patients reported that they had great difficulty in maintaining a healthy diet especially during the holidays or going out to eat with other people: changing unhealthy dietary habits is very challenging for ACS patients if their relatives and close friends do not change their behavior as well. Finally, dietary adherence in ACS patients may be very challenging because it may require multiple dietary changes, from reducing sodium and saturated fat intake to increasing consumption of fish, vegetables and fruits. Thus, dietary recommendations after ACS are generally too complex and hard to recall and they could lead to confusion and suboptimal diet change [35].

The most maladaptive clusters were defined as those in which two unhealthy behaviors co-occur in the majority of patients. Particularly, before the acute coronary event these kinds of clusters highlighted an association between low levels of physical activity and smoking behavior (Cluster 2) and an association between low adherence to the Mediterranean diet and smoking (Cluster 1). At six months after the acute event, the most maladaptive lifestyle is characterized by the co-occurrence of unhealthy diet and inappropriate physical activity (Cluster 3). The same lifestyle profile was also identified at twelve months after the coronary event (Cluster 2). Clusters with all smoking members are present at each wave, even though smoking is the behavior presenting the largest improvement in the whole group (90% of patients are non-smokers at T2), suggesting a greater ease of change for this behavior compared to diet and physical activity [36]. By looking at the percentage of patients belonging to these most maladaptive lifestyle profiles at each wave, after an initial decrease from baseline to six months after, this proportion registered a slight increase from six to twelve months after the acute event, suggesting the difficulty patients have in maintaining a healthy lifestyle.

Looking at individual pathways of lifestyle change from baseline to T1 and from T1 to T2, both positive (i.e., in the direction of healthier behavior) and negative (i.e., in the direction of unhealthier behavior) pathways were detected. As expected, patients with a healthier profile, specifically members of Cluster 5 at baseline, tend to reemerge in a similar profile at a later time (Cluster 5 and 6 at T1, and Cluster 5 at T2) demonstrating their ability to further improve their behavior. In line with our expectations, several patients in the worst cluster at baseline (Cluster 1) improve their behavior (moving to Cluster 2), becoming more physically active and stopping smoking. However, they are unable to change their diet. This further confirms the difficulty in changing such a complex health related behavior and suggests the difficulty of changing a very compromised and risky behavioral profile. Only some of the patients in Cluster 2 further improve their behavior six months after the event, in terms of physical activity and smoking, moving to Cluster 3 at T2. Other patients move to the worst cluster at the final assessment, Cluster 2, in which none of the patients eats properly or is physically active. This result further confirms the difficulty that patients experience in maintaining the changes that they have reached, especially when the demands of change regard several aspects of their lifestyle. Cluster 4 in each wave is characterized by stably inappropriate physical activity. These people are less physically active six months after the acute coronary event and they still engage in little activity twelve months after. Interestingly, these patients are able to adhere better to a healthy diet and completely refrain from smoking. This suggests a specific difficulty in getting an adequate level of physical activity that could perhaps be explained by the older age of these patients.

Three pathways showed worsening in patients' lifestyle profile, disconfirming our hypothesis that patients belonging to unhealthier profiles were more likely to belong to healthier profiles at subsequent waves. Specifically, Cluster 3 at T0 to Cluster 1 at T1 reflect stable smoking behavior (all cluster members are smokers at both times) and a decrease in physical activity. This may find an explanation in the previous research of Cooper and colleagues highlighting patients' intense fear and concern in practicing physical activity [37–38]. Specifically, their results underlined that ACS patients view their shortness of breath during physical activity as a source of apprehension and something to be avoided because they associate this functional breathlessness with what they experienced during the acute coronary event. Finally, ACS patients generally view exercise as a source of worry and embarrassment because of the negative comparisons between themselves and fit people who attend gyms and exercise regularly.

The other two pathways are from Cluster 3 at T1, characterized by inappropriate diet and physical activity. Some of the patients move to Cluster 1 at T2, improving these two behaviors but start smoking again, whereas other patients move to Cluster 2, in which all patients avoid the Mediterranean diet but practice proper physical activity. These results further confirm the difficulty patients with a multi-problematic profile have to make changes.

By considering external criteria, partly in line with our hypotheses the results demonstrated one association between lifestyle profiles at twelve months after the acute coronary event and depression measured six months earlier, after controlling for gender and age. People in the cluster with all smoking patients at the final assessment were more depressed six months before than patients in the healthiest clusters. These results are consistent with previous empirical findings showing that smokers with high depressive symptoms are less likely to effectively quit smoking and maintain abstinence [39–40]. These findings also suggested that higher levels of depression six months after the acute coronary event are associated with subsequent unhealthiness and maladaptiveness of lifestyle six months later. This result is consistent with empirical evidence showing that patients with depression are less likely to follow recommendations to reduce lifestyle risk factors for cardiovascular disease and thus these patients are less likely to improve their unhealthy lifestyle [41]. However, higher levels of depression at baseline are not associated with subsequent unhealthiness or maladaptiveness of lifestyle six and twelve months later. We could speculate that the non-significance of difference in depression among clusters at these waves could be due to the recent experience of the acute coronary event. At baseline all patients were encountering significant psychological distress associated with the acute event that could confound possible differences in depression among lifestyle profiles. This explanation is coherent with previous empirical findings showing that depression is very common following a myocardial infarction [42].

However, differently from our hypothesis, anxiety symptoms are never associated with lifestyle profiles. As suggested by Farley and colleagues [19], further research should explore the impact of ACS anxiety on both CR attendance and behavioral change. In fact, while our study does not provide evidence of the association between anxiety and an unhealthy lifestyle profile, mixed and contrasting results of the positive [43] and negative [18–19] impact of anxiety on CR attendance and behavioral change have been previously reported.

Moreover, coherently with our hypothesis of finding an association between elevated HR and cluster membership at each wave, the most maladaptive lifestyle profile at T2 had many members with elevated HR who are, perhaps, more likely to be at risk of subsequent cardiovascular death and morbidity [21]. This latter result is congruent with previous empirical evidence of the detrimental effect of low physical activity, heavy smoking, and inadequate diet on elevated HR [22–23]. However, we found no link between HR and cluster membership at baseline or T1. Taken together, these two latter results may suggest a time cumulative effect of unhealthy cluster membership on HR.

The current study has several limitations that must be considered in interpreting our results. First, at T0, retrospective measures of unhealthy lifestyles were considered; patients were asked to report their conduct before the acute event. This approach may limit the reliability of the variables observed in measuring actual healthy behaviors. It is possible that patients may have overestimated or underestimated their real past healthy lifestyles. Second, we did not objectively collect or directly assess measures of diet, physical activity and cigarette smoking, but considered only self-reported behaviors, a methodology widely adopted in the medical and psychological literature [24, 26–27]. Nevertheless, the use of self-report measures makes it possible to obtain a valid and reliable proxy of the actual behavior; for example, a study assessing the validity of reported physical activity found that correlations between self-report and direct measures were generally moderate-to-strong, ranging from -0.71 to 0.96 [44].

Despite these limitations, the current study reports several original findings that suggest important implications for further research and practical applications in behavioral intervention for ACS patients. One of the most important outcomes is the strong interrelatedness between lifestyle risk factors. Like healthier individuals, some ACS patients are characterized by multiple unhealthy behaviors. Similar to results on healthy individuals [13, 45], our study demonstrated that ACS patients and smokers are more likely to have an inappropriate diet in that they consume more fat and eat fewer fruits and vegetables. Moreover, we found that a sedentary lifestyle is generally clustered with smoking and unhealthy diet, replicating the finding generally reported in the general population [46–47]. Moreover, the nearly high prevalence and individual stability of some of the unhealthier profiles suggests that patients with multiple lifestyle risk factors experience great difficulties in changing their unhealthy behaviors after the acute coronary event. Thus, we suggested promptly and accurately evaluating patients’ lifestyle upon admission for CR, in order to identify the possible co-occurrence of unhealthy behaviors and subsequently deliver an appropriate multi-target intervention program to effectively tackle this interrelatedness. In fact, as suggested by Burke [48], these interrelated, unhealthy behaviors are most effectively targeted by multimodal and multi-target interventions addressing wider-ranging improvement in lifestyles. Patients with multiple behavioral risk factors may warrant particular attention, as also suggested by the associations of unhealthier lifestyle clustering and dysfunctional individual pathways with depression and the HR. The association between higher levels of depression and subsequent unhealthiness and maladaptiveness of lifestyle suggests the need for an incisive and reliable screening of psychological distress in ACS patients in order to better support more depressed patients in effectively changing their multiple risk behavior.

In conclusion, the findings from the present study may be particularly relevant in terms of secondary prevention because focusing on the interrelation among unhealthy behaviors in patients at their first coronary event may lead to developing personalized secondary prevention programs that target multiple behaviors at a time. This type of program may have a strong impact on healthcare and clinical practice. As also effectively underlined in a recent meta-analysis by Prochaska and Prochaska [49], an accurate analysis of the co-occurrence and change of lifestyle risk factors is a prerequisite for implementing an effective multiple health behavior change intervention by shedding light on the range of mechanisms that may account for the synergic and multiplicative effects in behavioral clusters. Thus, as also suggested by these authors in their pioneering work, we believe that targeting change in multiple risk behaviors may have a beneficial impact on the improvement of ACS patients’ health status and on reducing the health care costs of ACS treatment.

## Supporting information

S1 Dataset Dataset of the study. (SAV)
